# Supplementary material for: Resolving the origins of secretory products and anthelmintic responses in a human parasitic nematode at single-cell resolution
Source: eLife. 2023 Jun 15;12:e83100. doi: 10.7554/eLife.83100 (PMC10319439; doi:10.7554/eLife.83100)
Supplement: Figure 1—source data 1. — Determining the total cell count in the suspension was difficult due to remnants of debris and small and variable sized cells within the suspension. The recommended tabletop automated cell counting system Countess II (Thermo Fisher, Waltham, MA) was unreliable in confirming total and viable cell counts, likely due to cell sizes below the lower threshold of the instrument and because the cells are in multiple focal planes within the chip. Fluorescent-activated cell sorting (FACS) was also used to estimate cell count and attempt enrichment of viable cells prior to loading the 10x Genomics. The majority of cells did not survive the FACS process and could not be recovered for input into the 10x Genomics Chromium controller. Estimating cell concentration was most reliable using a hemocytometer. Bpa, Brugia pahangi; Bma, Brugia malayi; mf, microfilariae. [file elife-83100-fig1-data1.docx]

date species stage measurement staining input total_cells viable_cells units notes

20201110 Bpa mf FACS draq5/dapi 300000 7505 1644

20201110 Bpa mf FACS draq5/dapi 120000000 17811 4536

20201110 Bpa mf CountessII draq5/dapi 300000 167000 8785 cells/mL pre-sort

20201110 Bpa mf CountessII draq5/dapi 120000000 1610000 29350 cells/mL pre-sort

20201110 Bpa mf CountessII draq5/dapi 300000 44000 0 cells/mL post-sort

20201110 Bpa mf CountessII draq5/dapi 120000000 88000 0 cells/mL post-sort

20201117 Bma mf FACS draq5/dapi 120000000 4040000 500000 cells/mL

20201117 Bma mf CountessII draq5/dapi 120000000 49800 49830 cells/mL post-sort

20201117 Bma mf hemocytometer NA 120000000 1870000 NA cells/mL

20201215 Bma mf CountessII NA 120000000 633000 NA cells/mL

20201215 Bma mf hemocytometer NA 120000000 2070000 NA cells/mL

20201215 Bma mf FACS draq5/dapi 120000000 669812 71292 cells/mL
